# The Frequency of Immunofluorescence Antinuclear Antibody Patterns and Extractable Nuclear Antigen: Experience From a Large Laboratory in Pakistan

**DOI:** 10.7759/cureus.33343

**Published:** 2023-01-04

**Authors:** Kiran Imran, Asif Loya, Maryam Hameed, Imran A Siddiqui, Umer N Sheikh

**Affiliations:** 1 Pathology, Shaukat Khanum Memorial Cancer Hospital and Research Centre, Lahore, PAK; 2 Histopathology, Shaukat Khanum Memorial Cancer Hospital and Research Centre, Lahore, PAK

**Keywords:** extractable nuclear antigen (ena), antinuclear antibodies, autoimmune disease, autoantibodies, immunofluorescence

## Abstract

Background

Autoimmune disorders have shown an increasing incidence in the last few years. The systemic response to the disorder is characterized by the expression of antinuclear antibody (ANA), which serves as the serological hallmark of autoimmunity. Its presence may indicate either a systemic autoimmune disease such as systemic lupus erythematosus (SLE), scleroderma, and polymyositis/dermatomyositis or an organ-specific condition such as autoimmune thyroiditis and hepatitis. The systemic response may vary from one individual to another in each population. Several specific autoantibodies are also found to be associated with specific rheumatic diseases.

Aim

We aim to report the frequency of ANA positivity, ANA immunofluorescence patterns, and the presence of extractable nuclear antigen (ENA) among the general Pakistani population from one of the largest laboratories in Pakistan.

Material and methods

A total of 1,966 blood samples from a random Pakistani population were included, who were referred by their physicians with suspicion of autoimmune disease. These blood samples were subjected to ANA testing by indirect immunofluorescence method, and subsequently, positive samples were further analyzed for ENA detection in the Section of Chemical Pathology, Department of Pathology at Shaukat Khanum Memorial Cancer Hospital and Research Centre, Lahore, Pakistan. An ANA titer of ≥1:80 was taken as positive. ANA was divided into subgroups based on titer: negative, weakly positive (titer of 1:80 or 1:160), moderately positive (titer of 1:320 or 1:640), and strongly positive (titer of ≥1:1280). Further, the frequency of ANA in male and female participants was studied in different age groups (2 to <10, 10 to <20, 20 to <30, 30 to <40, 40 to <50, 50 to <60, 60 to <70, 70 to <80, and 80+ years).

Results

This study included 1,966 participants, out of which 1,100 (55%) were ANA-positive at a titer of ≥1:80. Out of these ANA positives, the proportion of weakly positive (titer of 1:80 or 1:160), moderately positive (titer of 1:320 or 1:640), and strongly positive (titer of ≥1:1280) were 48.7%, 2.6%, and 4.2%, respectively. The ages ranged from two to 91 years, with a mean age of 43.64 ± 17.4 years. Females (75.5%) showed predominance over males (24.5%) in all age groups, with a ratio of 3:1. The age group in which most ANA positivity was found was 30 to <40 years. Among 1,100 ANA-positive sera, 383 (34.8%) participants tested positive for at least one out of 15 ENA. The most frequent autoantibodies noticed were anti-recombinant Ro52 (Ro52) (19.8%), anti-Sjogren’s syndrome type A (SSA) (17.2%), and anti-ribonucleoprotein (RNP) (13.3%). The most prevalent ANA patterns were nuclear homogeneous (27.7%), followed by nuclear speckled (26.5%).

Conclusion

The frequency of ANA positivity is high in the Pakistani population and differs in different sex and age groups.

## Introduction

The incidence of autoimmune diseases varies worldwide mainly due to differences and variations in immunologic status and individual response to disease among individuals in each population. Multiple etiologic factors may be responsible for the causation of these disorders, such as genetic factors or environmental factors including stress, age, sex hormones, or infection exposure [1]. Research has proven the autoaggression of the body’s immune system via the production of autoantibodies against self-antigens in the affected individuals [2]. These antibodies known as antinuclear antibodies (ANAs) serve as the fundamental biomarker in the identification, diagnosis, and monitoring of various autoimmune disorders [3]. A range of antibodies against the nucleus, nucleolus, cytoplasm, and mitotic cellular apparatus can be detected by ANA assay [4]. This assay is used as an initial screening method, where antibodies are detected by the indirect immunofluorescence method on human epithelial 2 (HEp-2) cells. The presence of ANA is an entry criterion for systemic lupus erythematosus (SLE) in a recent classification [5]. In children with juvenile idiopathic arthritis, ANA serves as a prognostic marker for uveitis [6]. In addition, for the diagnosis of autoimmune hepatitis, ANA positivity and titers are included in the criteria [7].

Despite the test’s great clinical utility and sensitivity, this test should be followed by an anti-extractable nuclear antigen (ENA) detection for the confirmation of diagnosis. This is due to the presence of low levels of ANA in 30% of healthy individuals [8]. ANA positivity solely may not essentially indicate the presence of a disease [8]. A population-based study from China reported that 6% of healthy individuals had ANA positivity with a titer of 1:320 [9], while 35% of healthy individuals showed ANA positivity in a study conducted in Mexico (titer of 1:40) [10]. Moreover, the presence of ANA is nonspecific and is expressed in certain non-autoimmune conditions such as cancer, chronic infections, cardiovascular diseases, and the use of certain medications [11,12]. The increasing levels of inflammation in affected organs can be one of the possible reasons. ANA immunofluorescence patterns based on the intranuclear distribution of the antigen can be subdivided into nucleolar, homogeneous, centromere, speckled, nuclear dot, nuclear membrane, mixed, etc. [13]. Follow-up testing by ENA profile plays a vital role in the evaluation of proteins within the nucleus that are recognized by antibodies. These include immunoglobulin class IgG to different antigens, namely, Smith (Sm) antigen, ribonucleoprotein (RNP) 70, Sjogren’s syndrome type A (SSA), recombinant Ro52 (Ro52), Sjogren’s syndrome type B (SSB), scleroderma/topoisomerase (Scl-70), histidyl-tRNA (Jo-1), anticentromere (CB), histones (his), proliferating cell nuclear antigen (PCNA), double-stranded DNA (dsDNA), nucleosomes (Nuc), ribosomal protein (Rib), anti-mitochondrial (M2) antibody, and polymyositis/scleroderma (PM-Scl). The presence of one or more of the autoantibodies against these antigens, along with specific ANA immunofluorescence patterns, aids in the diagnosis of certain autoimmune diseases. The present study aims to determine ANA positivity, the frequency of different ANA immunofluorescence patterns, and the frequency of ENA in the local population of Pakistan.

## Materials and methods

This retrospective, cross-sectional study aims to include blood samples performed during one year (January 1, 2021-December 31, 2021), irrespective of age and gender. A total of 1,966 blood samples of individuals who were referred to our laboratory by their physicians for ANA immunofluorescence detection were included in the study. Among these ANA-positive sera, further evaluation was done for extractable nuclear antigens in 383 sera, which tested positive for at least one out of 15 extractable nuclear antigens. Blood specimen (2-4 mL) was collected in a clot activator (red cap) vacuum collection tube, which was centrifuged at 4,500 rpm for five minutes.

ANA detection by immunofluorescence technique

An indirect immunofluorescence test using human epithelial cells and a primate liver is the gold standard for the detection of antinuclear antibodies, due to its high sensitivity and specificity. In our center, the assessment of ANA patterns and titers was carried out with AESKU.DIAGNOSTICS Gmbh (Wendelsheim, Germany), which is a semiautomated high-throughput system. The kit consists of glass microscope slides that are coated with tissue sections or HEp-2 cells. HEp-2 is a human epithelium cell, cultivated from the tissue of a patient suffering from carcinoma of the larynx. After the dilution of samples, conjugation with fluorescein-labeled antihuman antibody conjugate was done. A specific green-colored, fluorescent staining pattern of antigen-antibody complexes was visualized with the aid of a fluorescent microscope under 10× and 40× objectives. The slides were evaluated in comparison with positive and negative controls provided in the manufacturer kit. Qualified laboratory consultants assessed these slides. A titer of ≥1:80 was used as a cutoff for ANA positivity as recommended by the manufacturer of this HEp assay. ANA was divided into subgroups based on titer: negative, weakly positive (titer of 1:80 or 1:160), moderately positive (titer of 1:320 or 1:640), and strongly positive (titer of ≥1:1280). Further, the frequency of ANA in male and female participants was studied in different age groups (2 to <10, 10 to <20, 20 to <30, 30 to <40, 40 to <50, 50 to <60, 60 to <70, 70 to <80, and 80+ years).

ENA

ANA-positive sera (titer of ≥1:80) were further analyzed by specific second-step autoantibody assays for further confirmation. The qualitative determination of human autoantibodies of the immunoglobulin class IgG in serum or plasma was carried out using the EUROLINE® Immunoblot (EUROIMMUN Medizinische Labordiagnostika AG, Lübeck, Germany) for specific autoantibodies, which include anti-Smith (Sm) antigen, anti-ribonucleoprotein (RNP) 70, anti-Sjogren’s syndrome type A (SSA), anti-recombinant Ro52 (Ro52), anti-Sjogren’s syndrome type B (SSB), anti-scleroderma/topoisomerase (Scl-70), anti-histidyl-tRNA (Jo1), anticentromere (CB), anti-histones (his), anti-proliferating cell nuclear antigen (PCNA), anti-double-stranded DNA (dsDNA), anti-nucleosomes (Nuc), anti-ribosomal protein (Rib), anti-mitochondrial (M2) antibody, and anti-polymyositis/scleroderma (PM-Scl). Diluted patient samples were incubated with the immunoblot strips. The specific IgG antibodies were bound to corresponding antigenic sites in the positive samples. Bound antibodies were detected after a second incubation using an enzyme-labeled antihuman IgG (enzyme conjugate), which catalyzed a color reaction. The signal intensity of more than or equal to 11 (medium) was considered positive, whereas borderline positives were excluded.

These autoantibodies were studied in ANA-positive male and female individuals and also in different age groups (2 to <10, 10 to <20, 20 to <30, 30 to <40, 40 to <50, 50 to <60, 60 to <70, 70 to <80, and 80+ years).

Data analysis

Data were analyzed using the Statistical Package for the Social Sciences (SPSS) version 21 (IBM SPSS Statistics, Armonk, NY, USA). For continuous data, a t-test was applied, while a chi-squared test was done for categorical data. A p-value of less than 0.05 was taken as significant.

## Results

Of the 1,966 participants, 55% (1,100) were found to be ANA-positive (titer of ≥1:80). Out of these ANA positives, the proportion of weakly positive (titer of 1:80 or 1:160), moderately positive (titer of 1:320 or 1:640), and strongly positive (titer of ≥1:1280) were 48.7%, 2.6%, and 4.2%, respectively. The ages ranged from two to 91 years, with a mean age of 43.64 ± 17.4 years in both males and females. Females (75.5%) showed predominance over males (24.5%) in all age groups in a ratio of 3:1. In our study, ANA positivity was more prevalent in the age group of 30 to <40 years. The frequency of ANA in male and female participants with respect to age groups is shown in Table 1.

**Table 1:** Frequency of antinuclear antibody among male and female participants in various age groups ANA: antinuclear antibody

Age group (years)	Male	Female	P value
2 to <10	11/31	8/12	0.065
10 to <20	18/65	45/91	0.002
20 to <30	35/98	129/223	<0.001
30 to <40	43/94	191/328	0.032
40 to <50	44/93	157/274	0.095
50 to <60	41/81	144/227	0.043
60 to <70	42/78	114/164	0.017
70 to <80	29/40	34/49	0.748
80+	7/8	8/10	0.671
Total	270/588	830/1,378	

There was a statistical difference noted between male and female individuals in the age groups of 10 to <20, 20 to <30, 30 to <40, 50 to <60, and 60 to <70 years. However, no statistically significant difference was noted among male and female individuals of age groups <10, 40 to <50, and >70 years (P < 0.05).

Among 1,100 ANA-positive samples, 383 (34.8%) participants tested positive for at least one out of 15 ENA. ANA-positive sera were divided into three age groups (<20, 21-49, and >50 years). However, no significant statistical difference was found among these groups (Table 2).

**Table 2:** Distribution of specific autoantibodies in positive sera in various age groups RNP: ribonucleoprotein, Sm: Smith, SSA: Sjogren’s syndrome type A, Ro52: recombinant Ro52, SSB: Sjogren’s syndrome type B, Jo1: histidyl-tRNA, Scl-70: scleroderma/topoisomerase, CB: centromere B, dsDNA: double-stranded DNA, histones: his, PCNA: proliferating cell nuclear antigen, Nuc: nucleosomes, Rib: ribosomal protein, M2: mitochondrial antigen, PM/Scl: polymyositis/scleroderma

Autoantibodies		Positive cases		
	<20 years	21-49 years	>50 years	P value
Anti-RNP	3	38	10	0.029
Anti-Sm antigen	3	14	8	0.785
Anti-SSA	5	41	20	0.364
Anti-Ro52	7	48	21	0.630
Anti-SSB	1	9	6	0.945
Anti-Jo1	1	11	4	0.592
Anti-Scl-70	2	7	0	0.046
Anti-CB	0	1	4	0.111
Anti-dsDNA	2	10	3	0.395
Anti-his	1	17	8	0.558
Anti-PCNA	0	11	6	0.439
Anti-Nuc	3	12	1	0.028
Anti-Rib	2	5	1	0.123
Anti-M2	1	10	6	0.921
Anti-PM/Scl	2	10	8	0.812

These ANA-positive sera were divided into male and female groups. The most frequent autoantibodies noticed were anti-Ro52 (19.8%), anti-SSA (17.2%), and anti-RNP (13.3%), whereas anti-CB (1.3%), anti-Rib (2.08%), and anti-Scl-70 (2.3%) were the least frequent (Table 3).

**Table 3:** Frequency of autoantibodies by gender in positive sera RNP: ribonucleoprotein, Sm: Smith, SSA: Sjogren’s syndrome type A, Ro52: recombinant Ro52, SSB: Sjogren’s syndrome type B, Jo1: histidyl-tRNA, Scl-70: scleroderma/topoisomerase, CB: centromere B, dsDNA: double-stranded DNA, histones: his, PCNA: proliferating cell nuclear antigen, Nuc: nucleosomes, Rib: ribosomal protein, M2: mitochondrial antigen, PM/Scl: polymyositis/scleroderma

Autoantibodies	Male	Female	Frequency	P value
Anti-RNP	15	36	13.3	0.774
Anti-Sm	7	18	6.5	0.953
Anti-SSA	8	58	17.2	0.003
Anti-Ro52	11	65	19.8	0.005
Anti-SSB	2	14	4.17	0.163
Anti-Jo1	6	10	4.1	0.334
Anti-Scl-70	2	7	2.3	0.707
Anti-CB	1	4	1.3	0.717
Anti-dsDNA	2	13	3.9	0.216
Anti-his	5	21	6.7	0.313
Anti-PCNA	3	14	4.4	0.332
Anti-Nuc	3	13	4.17	0.574
Anti-Rib	0	8	2.08	0.078
Anti-M2	0	17	4.43	0.009
Anti-PM-Scl	5	15	5.2	0.798

 In our study, the most prevalent ANA patterns were found to be nuclear homogeneous (27.7%), followed by nuclear speckled (26.5%), as shown in Table 4.

**Table 4:** Distribution of ANA patterns ANA: antinuclear antibody, DFS: dense fine speckled, NuMa: nuclear mitotic apparatus

Pattern	Number	%
Nuclear		
Nuclear homogeneous	300	27.2
Nuclear speckled	292	26.5
Nuclear dotted	1	0.09
Nuclear DFS	13	1.18
Nuclear nucleolar	121	11
Coarse speckled	202	18.3
Nuclear centromere	10	0.9
Cytoplasmic ANA	1	0.09
Cytoplasmic speckled	94	8.5
Cytoplasmic fibrillar	3	0.27
Cytoplasmic filamentous	1	0.09
Cytoplasmic Golgi	1	0.09
Cytoplasmic rods and rings	1	0.09
Mitotic		
Mitotic spindle	14	1.27
Mitotic intercellular bridge	1	0.09
Mitotic centrosome	1	0.09
Mitotic chromosomal	1	0.09
Mitotic NuMa	4	0.36
Mixed	39	3.5
Total	1,100	100

## Discussion

ANA immunofluorescence is among the first-line tests in the diagnostic workup of an autoimmune disorder. Laboratory, especially in a developing country such as Pakistan, requires a reliable, affordable, accurate, and specific screening test. This assay is not widely available in most of the laboratories in Pakistan, except for a few laboratories, due to the cost and requirement of technical expertise and trained laboratory personnel. The indirect immunofluorescence technique on the HEp-2 substrate is the preferred method for the detection of ANA. This detects antibodies to intracellular components, because of which a specific staining pattern is produced [14]. These ANA patterns are highly specific and play a crucial role in diagnosing certain autoimmune disorders along with antibody titers.

Several studies related to ANA prevalence worldwide have been published, but insufficient research has been conducted in this field, especially in our part of the world. The current study is hospital-based and, to our knowledge, the first of its kind conducted in Pakistan. The ANA test reports of 1,966 participants were included in this study. The ANA positivity status was determined with the help of indirect immunofluorescence on HEp-2 cell lines and primate liver. Out of these 1,966 participants, 1,100 showed ANA positivity (55.9%) at a titer of ≥1:80. The positivity rate detected in our study is high as compared to the data published in other studies. A possible explanation for this can be that as per manufacturer recommendations, we included weak/low positives in this study at a cutoff of ≥1:80. The presence of low titers may not necessarily be clinically significant, in contrast to high titers, which are more likely to be associated with the presence or development of autoimmune disease. The positivity in our study may not actually reflect the true estimate of the autoimmune disease, but it provides insight into the frequency of ANA in a random Pakistani population. Few similar studies have been conducted in India by Gupta et al. [15] from Rajpur, Sebastian et al. [16] from Bangalore, and Minz et al. [17] from Chandigarh, which showed ANA prevalence of 33%, 38.2%, and 18.9%, respectively. Different studies from other countries such as Japan by Hayashi et al. (9.5%) [18], Turkey by Mengeloglu et al. (15.8%) [19] and Sener et al. (21.9%) [20], and Belgium by Peene et al. (23.5%) [21] showed lesser positivity compared to our study. However, Akmatov et al. [22] from Germany (titer of ≥1:80) and Prapinjumrune et al. [23] from Thailand reported frequencies of 33% and 39.6%, respectively, which were comparable with our study. Guo et al. [9] from China and Satoh et al. [24] from the USA documented 5.9% and 13.8% positivity, respectively, which contrasted with our findings.

In this study, female predominance was found in all age groups, which was similar to the data published worldwide, where a higher ANA positivity rate in females has already been established. Parks et al. [25] stated that estrogen is a modifier of autoimmunity, and childbearing may have a role in initial antigen stimulation or reducing tolerance to self-antigens. The mean age in our study was 43.64 (±17.43). However, there was a difference in mean age worldwide. Studies conducted by Satoh et al. [24] and Prapinjumrune et al. [23] reported high prevalence in the older age group, while Guo et al. [9], Minz et al. [17], and Mengeloglu et al. [19] reported prevalence in 32, 42, and 43 years, respectively. Few conditions such as infections and rheumatic disorders are more common in teenagers, while neurological manifestations are common in old age. Additionally, in the older age group, the presence of ANA may be due to a senile condition [15].

In our study, anti-Ro52 (19.8%) and anti-SSA (17.2%) were the most prevalent autoantibodies, which were consistent with the findings in Chinese and Japanese studies [9,18]. The presence of anti-Ro52 and anti-SSA serves as a crucial marker for systemic lupus erythematosus (SLE), Sjogren’s syndrome, myositis, systemic sclerosis, and primary biliary cholangitis [26]. The positivity rate of anti-RNP detected was 13.3% in our study, and anti-CB, anti-Rib, and anti-Scl-70 were the least detected. Coincidentally, studies from China and Japan also reported anti-Scl-70 to be the least prevalent antibody [9,18].

Among the nuclear patterns, the most frequently detected were the nuclear homogeneous and nuclear speckled, which agrees with the data published worldwide. These patterns were positively associated with SLE as stated by Minz et al. [17]. In our study, we also identified a few uncommon (mixed/undefined) patterns, which are mostly thought of no clinical significance. However, Sener et al. [20] and Prapinjumrune et al. [23] revealed that these rare ANA patterns are not only present in significant numbers but are also associated with certain diseases such as chronic hepatic conditions and carcinoma. An extensive study was also conducted by Vermeersch et al. [27] on rare ANA patterns, which revealed the association of nuclear envelope and nuclear dot patterns with autoimmune hepatitis and hepatic and colon carcinoma. Similarly, cytoplasmic patterns were considered insignificant, but they might be associated with certain undetected conditions as stated by Prapinjumrune et al. [23].

There are certain limitations in this study. It is a cross-sectional study, due to which factors responsible for the causation of autoimmune diseases cannot be identified or studied. It is a pilot study with data not taking into consideration various factors such as genetic predisposition, clinical diagnosis, metabolic disorders, cardiovascular diseases, occupation, or biochemical factors. An extensive and detailed large-scale study is required in the future, especially from Pakistan, to determine the relationship of ANA with various etiologic and biochemical factors. However, the strong point of this study is that it provides an overview of ANA positivity and autoimmunity status, especially in our part of the world, where despite the heavy disease burden, data is scarce.

## Conclusions

The frequency of ANA positivity is high in the Pakistani population and differs in different age and gender groups. ANA positivity was more prevalent in females, especially in the reproductive age group. Nuclear homogeneous and nuclear speckled were the most frequently detected patterns; however, unusual (mixed/undefined) patterns were also identified, which provides an area for further research.
